# Pediatric parkinsonism: clinical review and a proposed clinical algorithm

**DOI:** 10.3389/fneur.2026.1788936

**Published:** 2026-07-07

**Authors:** Valentina Naranjo-Lobo, María José Hidalgo-Bravo, Daniela Munoz-Chesta, Mónica Troncoso-Schifferli

**Affiliations:** 1 Movement Disorders Unit, Department of Pediatric Neuropsychiatry, Hospital Clínico San Borja Arriarán, Santiago, Chile; 2 Department of Pediatrics, Faculty of Medicine, Central Campus, University of Chile, Santiago, Chile

**Keywords:** dopaminergic dysfunction, genetic movement disorders, inborn errors of metabolism, juvenile parkinsonism, neurodevelopment, parkinsonism-dystonia, pediatric parkinsonism

## Abstract

Pediatric parkinsonism (PP) is a rare but disabling movement disorder characterized by bradykinesia and rigidity, with tremor being less frequent. Additional features include gait disturbances and impaired postural reflexes. In younger children, hypotonia is often a predominant sign. Clinical expression evolves with neurodevelopment and is frequently associated with other movement abnormalities, such as dystonia. The condition may arise from a variety of pathophysiological mechanisms and multiple etiologies, with genetic causes being particularly prominent. A classification system has been proposed based on age of onset, clinical features, prognosis and etiology, comprising: Developmental parkinsonism, Infantile degenerative parkinsonism, Parkinsonism in the setting of postnatal neurodevelopmental disorders, Parkinsonism associated with multisystemic brain diseases, Juvenile parkinsonism and dystonia-parkinsonism and Acquired parkinsonism. A specific assessment tool—the Infantile Parkinsonism-Dystonia Rating Scale—has recently been developed and primarily validated to quantify severity, monitor disease progression and assess treatment response. This review aims to provide a comprehensive overview of the clinical features, pathophysiology, etiologies and treatment approaches of pediatric parkinsonism. In addition, we propose a diagnostic algorithm to support clinical decision-making.

## 1 Introduction

Pediatric parkinsonism (PP) is a rare hypokinetic–rigid syndrome characterized by bradykinesia, rigidity, and hypokinesia. Resting tremor is less frequent than in adults. In younger children, hypotonia is often a prominent feature, whereas in older children gait disturbances and impaired postural reflexes are more prevalent. The syndrome is frequently incomplete and clinically complex, and its expression may evolve over time in relation to neurodevelopment. It is commonly associated with additional neurological manifestations, including dystonia, global developmental delay, epilepsy, neurodevelopmental disorders, and oculogyric crises, among others (1).

Epidemiological information on the frequency and age distribution of parkinsonism is limited. Available data from the United States indicate that the incidence of early-onset parkinsonism increases with age, from 0.8 per 100,000 person-years in those aged 0–29 years to 3.0 per 100,000 person-years in individuals aged 30–49 years (2).

According to the age at clinical onset, parkinsonism can be classified as follows:

- juvenile parkinsonism (JP), defined as symptom onset before 21 years of age. Since symptom onset may occur as early as the neonatal period, the terms “pediatric parkinsonism” or “infantile parkinsonism” are also used to refer to this age group.;
- early-onset or young-adult–onset parkinsonism, when onset occurs between 21 and 40 years of age (with some authors extending this definition up to 50 years);
- late-onset parkinsonism, when symptoms begin after 50 years of age (1, 3).

PP arises from disruption of dopaminergic neurotransmission through mechanisms that differ substantially from those underlying adult Parkinson’s disease (PD). Rather than *α*-synuclein aggregation, pediatric pathophysiological mechanisms most commonly involve defects in dopamine synthesis and transport, mitochondrial dysfunction, lysosomal and endosomal trafficking abnormalities, metal accumulation, impairments in synaptic vesicle recycling, and neuroinflammatory or toxic injury, among others (3, 4).

The etiology is predominantly genetic, with more than 70 genes described, and early recognition is crucial, as some forms are potentially treatable. Furthermore, PP encompasses a group of conditions in which timely recognition and appropriate intervention may lead to substantial clinical improvement and, in selected etiologies, even complete reversal of symptoms, allowing normal or near-normal neurodevelopment (4, 5).

However, PP remains an underrecognized entity, owing to its rarity, marked etiological and pathophysiological heterogeneity, and clinical features that differ substantially from those of adult Parkinson’s disease. These factors may hinder early diagnosis and delay access to targeted therapies, resulting in missed opportunities to significantly improve outcomes.

This review aims to provide a comprehensive overview of pediatric parkinsonism, including its clinical presentation, pathophysiological mechanisms, major genetic and acquired etiologies, diagnostic approach, and current treatment options. We also propose a practical diagnostic algorithm to facilitate early recognition and etiological evaluation, with particular emphasis on potentially treatable disorders and the importance of timely diagnosis.

## 2 Methods

A targeted narrative review was conducted to summarize current knowledge and insights on pediatric parkinsonism.

A structured literature search was performed in PubMed/MEDLINE as the primary database. The search strategy incorporated the following predefined terms: “(pediatric OR infantile OR juvenile) AND parkinsonism.” The search was restricted to studies published within the last 10 years, up to December 1, 2025. To ensure adequate contextualization, reference lists of relevant articles were manually screened, leading to the inclusion of additional studies published prior to 2015 when considered of historical or conceptual relevance.

Records were screened by title and abstract, followed by full-text review when deemed necessary according to predefined eligibility criteria. Inclusion criteria comprised original research articles, systematic reviews, narrative reviews, clinical guidelines, and high-quality case series focusing on pediatric populations. Studies addressing clinical features, pathophysiology, etiology, diagnostic approaches, and therapeutic strategies were prioritized.

Exclusion criteria included single-case reports with limited generalizability, articles not available in English or Spanish, and studies lacking direct clinical relevance to pediatric parkinsonism.

Data extraction was conducted qualitatively, focusing on key domains including clinical spectrum, etiologic classification, underlying pathophysiological mechanisms, diagnostic frameworks, and therapeutic approaches.

## 3 Classification of pediatric parkinsonism

PP is usually classified according to a diversity of criteria. The following are considered especially relevant for clinical practice.

### 3.1 Age-based classification

According to age at onset, PP can be classified as follows (1, 6):

- Infancy: onset between 1 month and 2 years of age.
- Childhood: onset between 3 and 12 years of age.
- Adolescence: onset between 13 and 21 years of age.

### 3.2 Clinical classification

Following Leuzzi et al. (4), and based on age at onset, clinical features, prognosis and etiology, PP can be classified into the following categories:

- Developmental parkinsonism: non-degenerative, characterized by hypokinesia, hypotonia and dystonia. Typically associated with neurotransmitter defects (TH, AADC, PTPS, SR).
- Infantile degenerative parkinsonism: caused by mutations in dopaminergic transporters (SLC6A3) or mitochondrial genes (WARS2). Clinically characterized by a progressive course and poor prognosis.
- Parkinsonism in the context of neurodevelopmental disorders: associated with mutations in MECP2, FOXG1, STXBP1, RAB39B, among others.
- Parkinsonism in multisystem brain diseases: including lysosomal disorders (Niemann–Pick type C, gangliosidoses), neurodegeneration with brain iron accumulation (NBIA) and leukodystrophies.
- Juvenile parkinsonism and dystonia-parkinsonism: classical monogenic forms (PARK2, PINK1, DJ1, SYNJ1, PLA2G6, FBXO7) presenting from late childhood to adolescence.
- Acquired parkinsonism: secondary to hypoxic, infectious/parainfectious, structural, autoimmune, toxin exposure, drug-induced, among other causes.

## 4 Pathophysiology of pediatric parkinsonism

The following section further discusses most of the pathophysiological mechanisms underlying PP, with particular emphasis on selected etiologies (see Table 1).

**Table 1:** Main pathophysiological mechanisms of pediatric parkinsonism.

Pathophysiological mechanisms of pediatric parkinsonism
Dopamine synthesis and transporter defects.
Mitochondrial dysfunction
Lysosomal dysfunction
Synaptic dysfunction
Metal accumulation
Acquired etiologies

### 4.1 Dopamine synthesis and transporter defects

Primary disorders of monoamine neurotransmission encompass defects affecting monoamine biosynthesis, catabolism, and transport.

#### 4.1.1 Dopamine synthesis defects

Primary neurotransmitter disorders are rare neurometabolic conditions characterized by genetic defects that impair neurotransmitter metabolism or transport. These involve abnormalities in enzymes critical for neurotransmitter biosynthetic pathways or in the essential cofactors required for their proper function (7).

Inherited disorders of neurotransmitters include defects in the biosynthesis of biogenic amines (dopamine, norepinephrine, epinephrine, and serotonin). These conditions are broadly categorized into those resulting from tetrahydrobiopterin (BH4) deficiency and those arising from primary enzymatic defects within the biosynthetic pathways (7).

**Figure 1:**
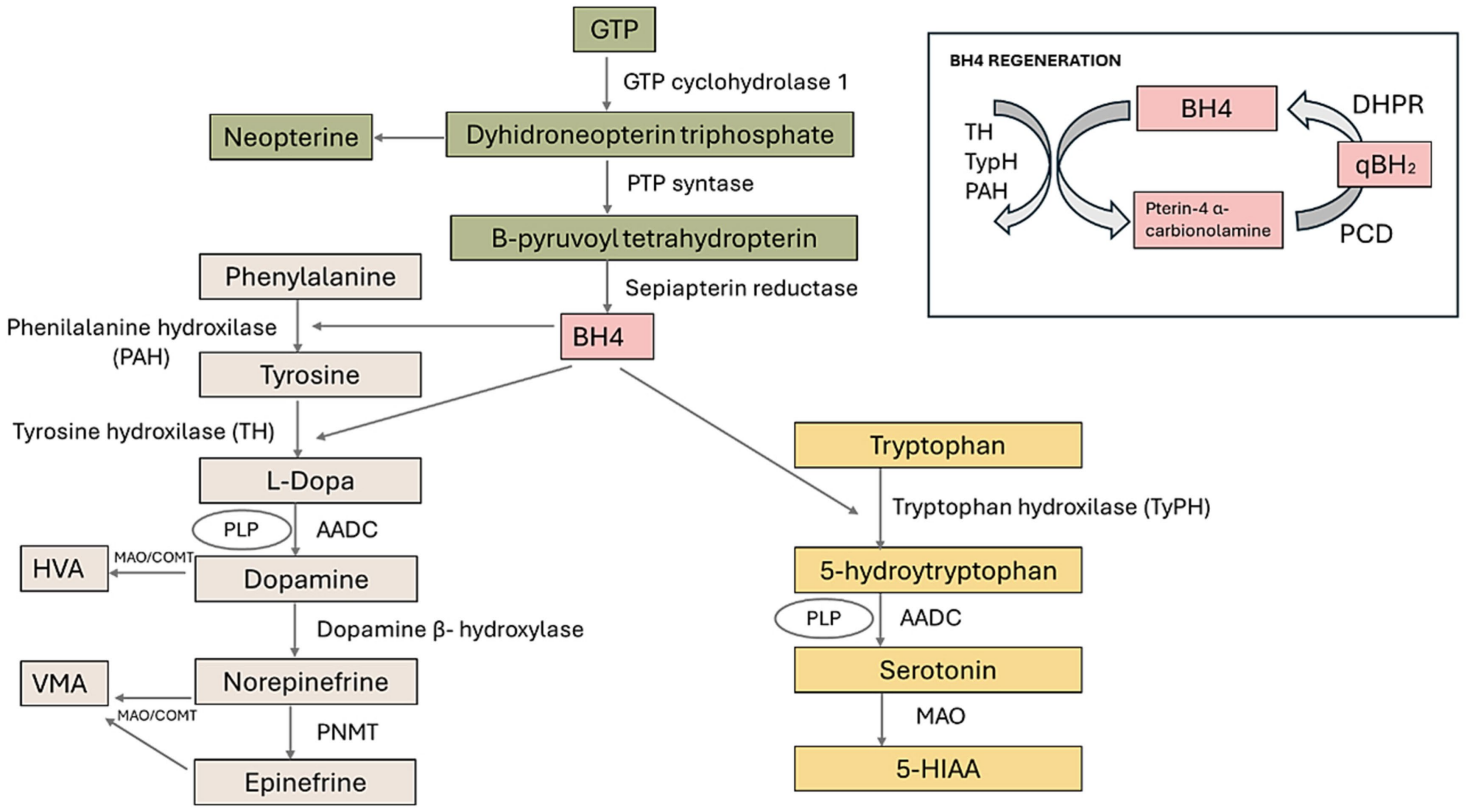
Tetrahydrobiopterin (BH4) metabolism and its role in monoamine neurotransmitter synthesis (7, 40). The diagram summarizes the biosynthesis and recycling of tetrahydrobiopterin (BH4), an essential cofactor in monoamine neurotransmitter metabolism. BH4 is required for the hydroxylation of phenylalanine to tyrosine, tyrosine to L-DOPA, and tryptophan to 5-hydroxytryptophan, which are critical steps in the synthesis of dopamine, norepinephrine, epinephrine, and serotonin. The BH4 regeneration cycle through quinonoid dihydrobiopterin (qBH2) and pterin-4α-carbinolamine is also shown. Homovanillic acid (HVA), vanillylmandelic acid (VMA), and 5-hydroxyindoleacetic acid (5-HIAA) are included as relevant biochemical markers in the diagnostic evaluation of neurotransmitter disorders associated with pediatric parkinsonism. PTPS: 6-pyruvoyltetrahydropterin synthase, DHPR: Dihydropteridine reductase, AADC: Aromatic L-amino acid decarboxylase, PLP: Pyridoxal 5′-phosphate, MAO: Monoamine oxidase, COMT: Catechol-O-methyltransferase, PCD: Pterin-4-carbinolamine dehydratase, Qbh2: Quinonoid dihydrobiopterin, PNMT: Phenylethanolamine N-methyltransferase.

BH4 is a critical cofactor for tryptophan hydroxylase (TypH) and tyrosine hydroxylase (TH), enabling the conversion of tryptophan to 5-hydroxytryptophan (5-HTP) and tyrosine to L-DOPA, respectively. These precursors are then decarboxylated by L-amino-acid-decarboxylase (AADC) to produce serotonin and dopamine, with dopamine subsequently converted into norepinephrine and epinephrine. Deficiencies in BH4 or AADC lead to chronically reduced monoamine neurotransmitter levels, whereas isolated TH deficiency affects only dopamine synthesis, with preservation of serotonin levels. BH4 biosynthesis may be compromised by defects in any of its four key enzymes— GTP cyclohydrolase I (GCH1), 6-pyruvoyltetrahydropterin synthase (PTPS), dihydropteridine reductase (DHPR), or sepiapterin reductase (SR)—resulting in decreased BH4 availability. Interestingly, BH4 is also an essential cofactor for phenylalanine hydroxylase (PAH), so hyperphenylalaninemia may be present in some patients with BH4 deficits (autosomal recessive (AR)GCH1, PTPS and DHPR deficiencies). Thus, these defects may be detected by neonatal screening for phenylketonuria (PKU) (7) (see Figure 1).

Inherited disorders of biogenic amine biosynthesis may exhibit a broad range of neurological manifestations and are typically associated with a non-degenerative disease course (4, 5). The most severe forms may result in neonatal death. Others severe infantile phenotypes typically present with early-onset encephalopathy, and parkinsonism as a key clinical feature. Additional neurological manifestations may include hypotonia, motor restlessness, feeding difficulties, motor delay, spasticity, oculogyric crises, dystonia, rubral-like tremor, and autonomic dysfunction. On the other hand, intermediate or milder phenotypes display varying degrees and combinations of neurological symptoms such as developmental delay, parkinsonism, autonomic dysfunction, spastic paraparesis, and additional movement disorders, particularly dystonia (5).

The key biochemical studies for identifying defects in biogenic amine biosynthesis are cerebrospinal fluid (CSF) analyses of neurotransmitter metabolites and pterins (5).

Neuroimaging findings are often normal, although delayed myelination can occasionally be observed. Additional reported features include symmetric calcifications of the lentiform nuclei and progressive brain atrophy (7).

Dopaminergic functional imaging (such Dopamine Transporter [DaT]-SPECT [DaTSCAN] or dopaminergic PET) is typically normal and can aid in distinguishing disorders with preserved nigrostriatal function—such as monoamine synthesis defects—from those characterized by nigrostriatal denervation (6).

#### 4.1.2 Dopamine transporter (DAT) defects

DAT is the main regulator of synaptic dopamine transmisión. It maintains homeostasis by mediating the reuptake of extracellular dopamine into presynaptic neurons, thereby controlling synaptic levels within the mesolimbic and nigrostriatal pathways (8).

Dopamine transporter deficiency syndrome (DTDS) results from impaired reuptake due to DAT dysfunction. *In vitro* studies of *SLC6A3* variants associated with DTDS demonstrate markedly reduced transporter activity, including impaired dopamine binding, defective trafficking to the cell surface, and abnormal post-translational glycosylation. Consequently, dopamine reuptake fails, leading to persistently elevated synaptic dopamine and increased CSF homovanillic acid (HVA). Also, depletion of intracellular dopamine and downregulation of TH activity through aberrant presynaptic D2 receptor feedback has been observed in DAT knockout mouse models (8).

The classical DTDS phenotype, associated with biallelic loss-of-function *SLC6A3* variants, typically emerges in early infancy. Motor development delay, hypotonia, irritability and feeding difficulties are key clinical features. This is followed by a hyperkinetic movement disorder —characterized by chorea, dystonia, ballismus, and orolingual dyskinesias— that progressively evolves into a severe parkinsonism–dystonia syndrome, ultimately leading to akinesia in late childhood or early adolescence. In addition, status dystonicus and ocular motor abnormalities (including oculogyric crises, ocular flutter, eyelid myoclonus, and impaired saccade initiation) may occur. Cognitive function is often relatively preserved despite profound motor impairment (8).

A milder, more slowly progressive DTDS phenotype has also been described in individuals with biallelic *SLC6A3* missense variants. These patients typically experience normal early neurodevelopment, and although later (often in the second decade of life) they present with tremor, progressive bradykinesia, dytonia, and variable tone (8).

Classical early-onset DTDS is associated with a characteristic CSF neurotransmitter profile marked by elevated HVA with normal 5-hydroxyindoleacetic acid (5-HIAA), resulting in an increased HVA:5-HIAA ratio of 5.0–13.0 (normal: 1.0–4.0) (8). Brain MRI is often normal or shows mild, nonspecific abnormalities such as delayed myelination, periventricular leukomalacia, or prominence of the external frontotemporal spaces. DaTSCAN typically reveals absent or markedly reduced tracer uptake in the basal nuclei (8).

### 4.2 Mitochondrial dysfunction

Mitochondrial dysfunction plays a relevant role in idiopathic, toxin-induced (e.g., 1-methyl-4-phenyl-1,2,3,6-tetrahydropyridine [MPTP]), and monogenic forms of Parkinson’s disease (e.g., PARK-*parkin*, PARK-*PINK1*, PARK-*DJ1*, PARK-*FBXO7*). In addition, parkinsonism has been described in several primary mitochondrial disorders (e.g., POLG-related disorders, Pyruvate carboxylase [PC] deficiency, Dynamin-related protein 1 [DLP1 disease], Complex III deficiency, Mitochondrial aminoacyl-tRNA synthetase deficiency, Complex I deficiency, tRNA-ILE deficiency) (3, 4).

#### 4.2.1 PINK1–Parkin and impaired mitophagy

Parkin and PTEN-induced kinase 1 (PINK1) are key enzymes involved in initiating mitophagy in response to oxidative stress. Within this process, PINK1 functions as the primary sensor of mitochondrial damage, while Parkin acts as the effector responsible for executing mitochondrial quality control (9).

When a mitochondrion becomes damaged, PINK1 accumulates on the outer mitochondrial membrane (OMM), where it phosphorylates both OMM proteins and the cytosolic E3 ubiquitin ligase Parkin, thereby activating it. Activated Parkin ubiquitinates multiple OMM proteins on the impaired organelle. The resulting ubiquitin chains recruit autophagy adaptors such as OPTN, NDP52, and TAX1BP1, which in turn attract the core autophagy machinery. This initiates autophagosome formation around the damaged organelle, ultimately leading to its degradation following fusion with lysosomes (9).

Loss of Parkin and PINK1 function leads to the accumulation of oxidative stress and cellular damage, particularly in cells with high mitochondrial activity, such as neurons (3).

PARK-*parkin* (*PARK2*), an autosomal recessive form of typical juvenile parkinsonism, accounts for approximately 10–20% of early-onset Parkinson’s disease, with a median age at onset of around 31 years (range 3–81 years) and juvenile presentation occurring in roughly 19% of cases (3).

Bradykinesia is the most frequently reported motor feature, followed by tremor—often more prominent in the lower limbs—rigidity, dystonia, and postural instability. Additional commonly described features include sleep benefit, diurnal fluctuations, and hyperreflexia. Atypical signs and cognitive impairment are uncommon, each reported in only about 3% of cases. Olfaction is often preserved. The clinical course is typically benign, with a slow progression (3).

Neuropathologically, PARK2 is marked by neuronal loss in the substantia nigra pars compacta (SNpc), with relative preservation of the dorsal tier and minimal or absent neuronal loss in the locus coeruleus, dorsal vagal nucleus, raphe nuclei, and nucleus basalis of Meynert. Lewy body pathology is absent in two-thirds of cases (3).

Brain MRI is usually unremarkable (6); however, presynaptic dopaminergic imaging typically demonstrates more severe and relatively symmetric striatal signal loss compared with non-parkin forms of early-onset Parkinson’s disease (3).

PARK-*PINK1* (*PARK6*) is the second most common genetic cause of early-onset Parkinson’s disease, representing approximately 2–7% of cases, with a median age at onset of 32 years (range 9–67 years) and juvenile onset reported in approximately 15%. It follows an autosomal recessive inheritance pattern. Clinically, it is almost indistinguishable from PARK-*parkin* in both presentation and disease course; however, some reports suggest a higher prevalence of non-motor symptoms, including an increased frequency of anosmia (3).

Underlying neuropathological knowledge remains limited. Brain MRI is typically normal, while functional imaging demonstrates relatively symmetric presynaptic nigrostriatal dopaminergic signal los (3).

#### 4.2.2 WARS2-related disease

Mitochondrial aminoacyl-tRNA synthetases (mt-ARSs) are essential enzymes required for mitochondrial protein translation (10). Pathogenic variants in *WARS2*, which encodes the mitochondrial tryptophanyl-tRNA synthetase responsible for aminoacylating tRNA(trp) with tryptophan (11), have been associated with a broad phenotypic spectrum encompassing both epilepsy and movement disorders (12).

Biallelic loss-of-function variants typically result in a WARS2-related epileptic phenotype, characterized by neonatal- or infantile-onset developmental and epileptic encephalopathy (DEE) or other seizure types. In contrast, individuals who are compound heterozygotes for a pathogenic *WARS2* variant and the hypomorphic allele c.37 T > G (p. Trp13Gly) more commonly present with a WARS2-related movement disorder, including early-onset levodopa-responsive parkinsonism/dystonia and progressive myoclonus-ataxia/hyperkinetic movement disorders (12).

Patients with WARS2-related movement disorders often have a normal brain MRI. However, in some individuals, patchy or nonspecific periventricular T2 hyperintensities, cerebellar atrophy, or varying degrees of global cerebral atrophy may be observed. Pallidal T2 hyperintensity has been reported in at least one case. Dopaminergic striatal pathway involvement is evident on DaTSCAN imaging, which typically shows reduced radiotracer uptake in the basal nuclei (12).

### 4.3 Lysosomal dysfunction

Parkinsonism has been described in several disorders associated with lysosomal dysfunction.

*ATP13A2*, for example, encodes a lysosomal P5-type ATPase involved in cellular manganese homeostasis. Pathogenic variants in this gene are associated with a broad phenotypic spectrum, including PARK-ATP13A2, hereditary spastic paraplegia, amyotrophic lateral sclerosis, and neuronal ceroid lipofuscinosis (3).

PARK-*ATP13A2* (Kufor–Rakeb syndrome) is an autosomal recessive form of atypical juvenile parkinsonism characterized by dystonia, oculomotor abnormalities (such as supranuclear gaze palsy, slowed saccades, and oculogyric crises), facial–faucial–finger mini-myoclonus, upper motor neuron signs, psychosis, and dementia. Approximately 80% of patients develop symptoms before the age of 20. The disease typically exhibits a progressive course (3). Brain MRI in Kufor–Rakeb syndrome typically demonstrates diffuse cerebral and cerebellar atrophy, with occasional evidence of iron accumulation in the basal ganglia (6). Functional imaging generally reveals striatal dopaminergic denervation (3).

In addition, several lysosomal storage disorders have been associated with juvenile parkinsonism, including Niemann–Pick disease type C (NPC), Gaucher disease types 2 and 3, gangliosidoses-GM1 type II, neuronal ceroid lipofuscinosis (*CLN2, CLN3, CLN6*), Lafora disease, and Chediak–Higashi syndrome. Although heterozygous mutations in the glucocerebrosidase (*GBA*) gene—responsible for Gaucher disease—represent an important risk factor for late-onset Parkinson’s disease, they have not been linked to juvenile parkinsonism (3, 4).

### 4.4 Synaptic dysfunction

Synaptic vesicle recycling is a core process in synaptic transmission. Clathrin-mediated endocytosis (CME) represents one of the principal mechanisms underlying this process. Auxilin, encoded by *DNAJC6*, is a neuronally expressed co-chaperone that plays a key role in CME (13). Auxilin cooperates with the heat shock cognate protein 70 (Hsc70) to mediate the uncoating of clathrin-coated vesicles, thereby enabling the regeneration of nascent synaptic vesicles. These vesicles are subsequently refilled with neurotransmitters and fuse with the presynaptic membrane to support successive rounds of neurotransmitter release (14).

PARK-*DNAJC6* is associated with two main clinical phenotypes: juvenile-onset parkinsonism and early-onset parkinsonism. The juvenile-onset form is the most common, with a median age at onset of parkinsonian symptoms of approximately 10 years (range 7–14 years). In most individuals, developmental delay, intellectual disability, seizures, spasticity, additional movement disorders (including dystonia and myoclonus), as well as neuropsychiatric features are present and often precede the onset of parkinsonism. The disease course is rapidly progressive, with loss of independent ambulation occurring by mid-adolescence (15).

Brain MRI is often unremarkable, although some individuals with juvenile-onset disease show mild to moderate generalized cerebral atrophy and, less commonly, cerebellar atrophy. Dopaminergic imaging with DaTSCAN typically demonstrates reduced or absent radiotracer uptake in the basal ganglia (15).

In addition, *SYNJ1* encodes synaptojanin 1 (SJ1), a synaptically-enriched phosphoinositide phosphatase. At presynaptic terminals, SJ1 is essential for clathrin uncoating during synaptic vesicle recycling by dephosphorylating phosphatidylinositol 4,5-bisphosphate [PI(4,5)P₂] at the plasma membrane and facilitating the dissociation of clathrin adaptor proteins (13).

*SYNJ1*-related disorders are clinically heterogeneous, ranging from early-onset typical or atypical parkinsonism (PARK-SYNJ1) to early-onset treatment-resistant seizures accompanied by severe neurodegenerative decline (16).

MRI findings range from normal to generalized cerebral atrophy, while functional imaging demonstrates a severe, bilateral nigrostriatal dopaminergic deficit (3, 16).

### 4.5 Metal accumulation

At least three clinical entities are relevant on this regard.

#### 4.5.1 Wilson’s disease (WD)

An inherited metabolic disorder, WD is caused by biallelic pathogenic variants in the *ATP7B* gene (17). *ATP7B* encodes a transmembrane copper-transporting ATPase 2 that fulfills two essential functions in hepatocytes: biliary copper excretion and incorporation of copper into apoceruloplasmin to form functional ceruloplasmin (3). Pathogenic variants in *ATP7B* impair copper homeostasis, leading to progressive copper accumulation in the liver, basal ganglia, cornea, and other organs (17).

Clinical manifestations typically emerge between adolescence and early adulthood. Initial presentation is hepatic in approximately 40% of patients, neurological in 40–50%, and predominantly psychiatric in around 10% (17).

The neurological phenotype is highly heterogeneous and commonly includes dysarthria (reported in up to 90% of cases), tremor (22–55%), dystonia (10–65%), parkinsonism (20–60%), and other movement disorders such as choreoathetosis. Parkinsonism in WD is characterized by hypomimia, hypophonia, micrographia, and shuffling or freezing gait. Symptoms are usually symmetric, although unilateral tremor may occur (17).

Ophthalmologic examination with slit-lamp evaluation may reveal Kayser–Fleischer rings, resulting from copper deposition in Descemet’s membrane of the cornea. Although often asymptomatic, their presence is diagnostically valuable, as Kayser–Fleischer rings are rarely absent in patients with neurological involvemen. Only approximately 10% of such patients have a normal slit-lamp examination (17).

Biochemical abnormalities typically include increased 24-h urinary copper excretion, elevated serum copper levels, and reduced serum ceruloplasmin concentrations (18).

Brain MRI in Wilson’s disease frequently demonstrates T2-weighted hyperintensities involving the basal ganglia, thalami, cerebellum, midbrain, and pons, accompanied by varying degrees of global cerebral atrophy. However, MRI findings may be normal in some patients with neurological involvement (3). Functional imaging may reveal abnormalities in both presynaptic and postsynaptic dopaminergic markers, a pattern that is relatively specific for Wilson’s disease, as most neurodegenerative parkinsonian syndromes typically involve either the presynaptic or the postsynaptic compartment alone (17).

Because Wilson’s disease is a treatable condition, all patients with juvenile parkinsonism or juvenile dystonia should be systematically screened for WD.

#### 4.5.2 Neurodegeneration with brain iron accumulation (NBIA)

NBIA comprises a heterogeneous group of rare genetic neurological disorders characterized by progressive iron deposition within the brain, predominantly affecting the basal ganglia (3). To date, pathogenic variants in more than 12 genes have been implicated, including *PANK2, PLA2G6, COASY, ATP13A2, CP, AP4M1, FA2H, CRAT, SCP2, C19orf12, DCAF17, GTPBP2, WDR45*, and *REPS1* (18). Inheritance is most commonly autosomal recessive, with notable exceptions such as neuroferritinopathy, which follows an autosomal dominant pattern, and *β*-propeller protein–associated neurodegeneration (BPAN), which is inherited in an X-linked dominant manner (3).

Clinically, NBIA disorders may present with early-onset, progressive parkinsonism–dystonia. Diagnostic suspicion is often raised by the presence of atypical neurological features, including pyramidal and upper motor neuron signs, peripheral neuropathy, chorea, tremor, early bulbar dysfunction, cognitive decline, psychiatric or behavioral disturbances, seizures, and abnormal ocular motor findings, among others (3, 18). In many cases, characteristic neuroimaging features provide key diagnostic clues and can strongly suggest the underlying NBIA subtype (18).

#### 4.5.3 Hypermanganesemia with dystonia (types 1 and 2)

Manganese is essential for normal brain function and metabolism, acting as a cofactor for numerous enzymes. However, its homeostasis must be tightly regulated, as both deficiency and excess are harmful. Manganese homeostasis in humans is maintained through a coordinated network of SoLute Carrier (SLC) transporters. SLC30A10 functions as a manganese efflux transporter expressed in hepatocytes and enterocytes, promoting manganese excretion into bile and the intestinal lumen. In contrast, SLC39A8 and SLC39A14 are manganese uptake transporters located on opposite membranes of polarized cells such as hepatocytes and enterocytes. SLC39A14, expressed on the basolateral side, shuttles manganese into hepatocytes and enterocytes for subsequent excretion via SLC30A10. SLC39A8, expressed on the apical membrane, is required for manganese uptake into the cell. Efflux of manganese from cells into the bloodstream at the basolateral membrane is thought to occur via ferroportin (FPN) (19).

Pathogenic variants in *SLC30A10* and *SLC39A14* lead to impaired biliary and intestinal manganese excretion, resulting in systemic metal accumulation and subsequent neurotoxicity. Multiple molecular pathways have been implicated in manganese-induced neuronal injury, including oxidative stress, mitochondrial dysfunction, disturbances in calcium homeostasis, and endoplasmic reticulum stress (19).

Hypermanganesemia with dystonia 1 (HMNDYT1) results from biallelic pathogenic variants in *SLC30A10* and presents as a childhood-onset dystonia–parkinsonism accompanied by systemic involvement, including chronic liver disease that may progress to cirrhosis, polycythemia, and iron depletion. In contrast, hypermanganesemia with dystonia 2 (HMNDYT2), caused by biallelic pathogenic variants in *SLC39A14*, is characterized by a predominantly neurological phenotype, with rapidly progressive childhood-onset dystonia–parkinsonism (19). The “cock-walk” gait is a distinctive dystonic gait pattern characterized by toe-walking with exaggerated high stepping, and is considered a hallmark of manganese overload, regardless of the underlying etiology (20).

Brain MRI may reveal characteristic signal abnormalities, including hyperintensity of the globus pallidus, striatum, dentate nucleus, cerebral white matter, and dorsal pons on T1-weighted images, with relative sparing of the ventral pons. Corresponding hypointensity on T2-weighted sequences may be present in the same regions (19).

If left untreated, both HMNDYT1 and HMNDYT2 follow a progressive neurodegenerative course, leading to severe neurodisability predominantly driven by disabling dystonia. In HMNDYT1, complications related to chronic liver disease further increase morbidity and contribute to premature mortality (19).

In addition, several acquired forms of manganese neurotoxicity have been described. The most common is manganism, which occurs in individuals with chronic manganese exposure, particularly in occupational settings such as mining, as well as in people who misuse drugs. Other causes include acquired hepatocerebral degeneration observed in patients with advanced liver cirrhosis, and manganese accumulation associated with total parenteral nutrition, especially in neonates. Parkinsonism, dystonia, and characteristic T1-weighted hyperintensities of the basal ganglia are shared features of both acquired and inherited disorders of manganese metabolism (20).

##### 4.5.3.1 Acquired parkinsonism

Drug-induced parkinsonism (DIP) is likely the most common cause of secondary parkinsonism in childhood (21) (see Table 2). It is typically caused by dopamine-blocking agents, including antipsychotics, antiemetics, and dopamine-depleting drugs, but has also been reported with other medications such as calcium channel blockers, captopril, antiepileptic drugs (including valproate and phenytoin), selective serotonin reuptake inhibitors (SSRIs), lithium, and chemotherapeutic agents (including vincristine, cytarabine, cyclophosphamide, ciclosporin, methotrexate, and vinca alkaloids), as well as isoniazid, amphotericin B, chloroquine, doxorubicin, and meperidine (3, 4, 21, 22). Clinically, DIP is often characterized by symmetric parkinsonism with symptom onset following exposure to the offending medication. Symptoms usually resolve within days to months after drug withdrawal. Persistence of parkinsonian features beyond 3 months should raise suspicion for tardive parkinsonism or an underlying genetic or neurodegenerative parkinsonian disorder (3). DaTscan may be useful in distinguishing DIP from degenerative forms of parkinsonism (23).

**Table 2:** Acquired etiologies of pediatric parkinsonism.

Category	Representative causes
Infectious/post-infectious	Human immunodeficiency virus (HIV); subacute sclerosing panencephalitis (SSPE); *Mycoplasma pneumoniae*; mumps; encephalitis lethargica; arboviral encephalitides (St. Louis, Japanese, Western equine); dengue virus; Epstein–Barr virus; varicella-zoster virus; coxsackie virus; congenital influenza exposure; prenatal exposure to maternal encephalitis lethargica
Immune-mediated	Systemic lupus erythematosus; anti-NMDA receptor encephalitis; autoimmune basal ganglia encephalitis (anti-D2 receptor)
Structural/brain injury	Cerebrovascular events; intracranial tumors (including brainstem, mesencephalic, and compressive lesions); hydrocephalus or ventriculoperitoneal shunt dysfunction; cerebral palsy; intracranial infections (abscess, tuberculoma); pineal region cysts/lesions; traumatic brain injury; cranial radiotherapy; osmotic demyelination (central pontine and extrapontine myelinolysis); hypoxic–ischemic injury (including prolonged hypotension or hypoxemia)
Toxic exposures	Manganese; carbon monoxide; cyanide; ethanol; organophosphates; MPTP; carbon disulfide; methanol; toluene; n-hexane; 3,4-methylenedioxymethamphetamine (MDMA)
Drug-induced	Dopamine receptor antagonists (e.g., haloperidol, risperidone, aripiprazole); antiemetics; monoamine-depleting agents (e.g., reserpine); chemotherapeutic agents (e.g., vincristine, cytarabine, cyclophosphamide, methotrexate, ciclosporin, doxorubicin); antiepileptic drugs (e.g., valproate, phenytoin); selective serotonin reuptake inhibitors (e.g., fluoxetine); calcium channel blockers; isoniazid; lithium; captopril; amphotericin B; chloroquine; opioids (e.g., meperidine)
Other systemic causes	Disorders of calcium metabolism (hypoparathyroidism, pseudohypoparathyroidism); post-anoxic states (including anaphylaxis); severe burns; homocystinuria; Chediak–Higashi syndrome

HIV, human immunodeficiency virus; SSPE, subacute sclerosing panencephalitis; NMDA, N-methyl-D-aspartate; D2R, dopamine D2 receptor.

Other causes of acquired parkinsonism in childhood include autoimmune disorders—such as anti-NMDA receptor encephalitis, systemic lupus erythematosus, and basal ganglia encephalitis associated with anti-D2 receptor antibodies—as well as infectious and post-infectious etiologies. Reported associations include dengue virus, *Mycoplasma pneumoniae*, Epstein–Barr virus, Western equine encephalitis virus, St. Louis encephalitis virus, Japanese encephalitis virus, varicella-zoster virus, coxsackie virus, human immunodeficiency virus, subacute sclerosing panencephalitis, mumps, congenital influenza infection, and cases described in children born to mothers with encephalitis lethargica (3, 4, 21, 22).

Structural causes of acquired parkinsonism include stroke, prolonged hypotension or hypoxemia, traumatic brain injury, space-occupying lesions or infections (such as abscesses and tuberculomas), mesencephalic tumors, extra-axial compressive tumors, pineal gland cysts, obstructive hydrocephalus, cranial radiotherapy, central pontine and extrapontine myelinolysis, and cerebral palsy (3, 4, 21, 22).

Toxin-induced parkinsonism is exceedingly rare in the pediatric population. Nevertheless, reported toxic exposures include carbon monoxide, organophosphates, cyanide, MPTP, carbon disulfide, 3,4-methylenedioxymethamphetamine (MDMA), toluene, methanol, *n*-hexane, ethanol, and manganese (3, 4, 21, 22).

## 5 Clinical features by age of onset

**Figure 2:**
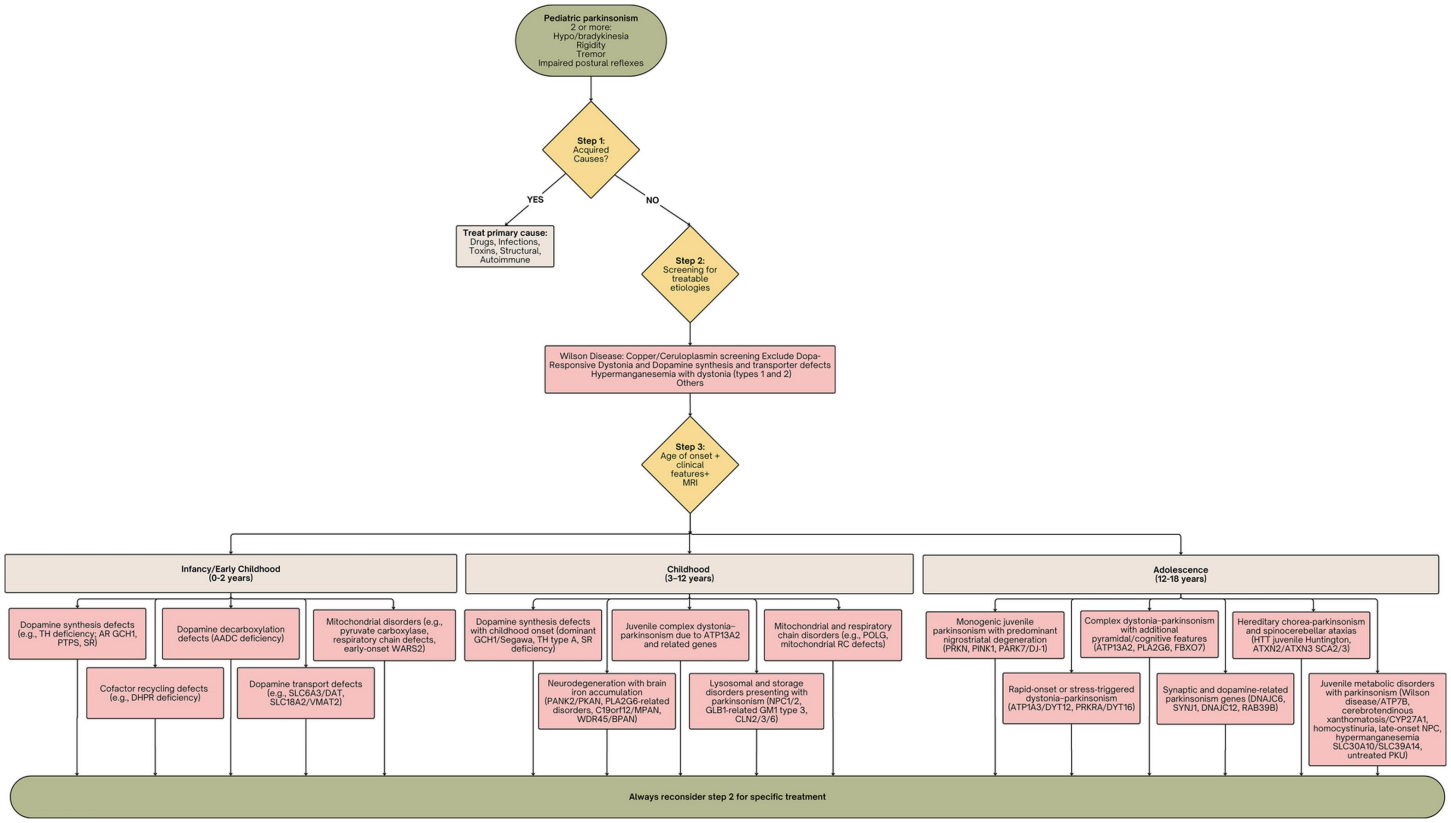
Proposed diagnostic algorithm for pediatric parkinsonism. This diagnostic algorithm summarizes a practical age-stratified approach to the etiological evaluation of pediatric parkinsonism.

The clinical manifestations and etiologies of PP vary markedly according to age at onset. In children younger than 2 years, parkinsonism-dystonia due to dopamine synthesis and transporter defects predominates; between 3 and 12 years, parkinsonism more often appears in association with other movement disorders and it is usually caused by degenerative conditions or complex metabolic disorders; finally, between 13 and 21 years, “pure” parkinsonism is more common and monogenic forms are predominant (*PRKN*, *PINK1*, *DJ-*1, etc.) (3–5) (See Figure 2).

### 5.1 Infancy: neonates to 2 years

Clinically, these patients typically present with a hypokinetic–rigid syndrome, characterized by marked hypokinesia, hypomimia, axial hypotonia, axial and orolingual dystonia with or without limb hypertonia, and motor developmental delay (see Table 3). A coarse tremor, not always present at rest, may also occur. In addition, oculogyric crises, ptosis, diurnal fluctuations, and autonomic features such as sweating, hypersalivation, and temperature instability may be observed (1, 20). The most frequent etiologies involve neurotransmitter disorders, including defects in dopamine and serotonin synthesis (such as TH, GCH1, AADC, PTPS, and SR deficiencies, among others) and DAT defects (8, 24–26).

**Table 3:** Infancy parkinsonism (0–2 years): Clinical features and Diagnostic workup (4, 5, 25, 26).

Etiologic group	Key clinical features	Core biomarkers	Typical imaging/response to treatment
Dopamine synthesis defects (e.g., TH deficiency; recessive GCH1, PTPS, SR)	Infantile hypokinetic–rigid syndrome with axial hypotonia, limb hypertonia, oculogyric crises and coarse tremor; in more severe forms, early encephalopathy with global developmental delay and diurnal fluctuation of dystonia.	Markedly reduced HVA in CSF with variable changes in other monoamines and pterins in TH deficiency; combined low HVA and 5-HIAA with abnormal CSF pterin profile, sometimes with hyperphenylalaninemia, in tetrahydrobiopterin-pathway disorders.	Brain MRI is usually normal or shows mild, non-specific atrophy. Most patients show an excellent to good response to low-dose levodopa (often combined with BH4, 5-HTP or MAO-B inhibitors), although dyskinesias may emerge with treatment.
Cofactor recycling defects (e.g., DHPR deficiency)	Early-onset hypotonia, with dystonia, seizures, motor delay and pyramidal signs, often within a broader encephalopathic picture.	Elevated phenylalanine with increased biopterins; low HVA and 5-HIAA in CSF indicating combined dopamine and serotonin deficiency.	MRI can reveal a leukoencephalopathy, sometimes with basal ganglia calcifications; Treatment combines levodopa, 5-HTP, BH4 and folate supplementation, with variable but often meaningful neurological improvement.
Dopamine decarboxylation defects (AADC deficiency)	Severe early hypotonia and hypokinesia, oculogyric crises, generalized dystonia, dysautonomia, sleep disturbance and profound developmental impairment.	Very low CSF HVA and 5-HIAA, elevated vanillactic acid and markedly increased 3-O-methyldopa; plasma/urine monoamine metabolites are also characteristic.	Structural MRI is normal or non-specific, while dopaminergic PET demonstrates absent striatal uptake. Clinical response to dopaminergic agonists, MAO-B inhibitors and pyridoxine is usually partial and robust improvement is restricted to a minority of cases.
Dopamine transport defects (e.g., SLC6A3/DAT, SLC18A2/VMAT2)	Infantile parkinsonism–dystonia with severe rigidity–akinesia, prominent dystonia and oculogyric crises; affected children often fail to achieve independent sitting and may show mood or autonomic disturbances in VMAT2 deficiency.	In DAT deficiency, CSF HVA/5-HIAA ratio is markedly increased with otherwise unremarkable peripheral monoamines; in VMAT2 deficiency, urinary monoamine metabolites are elevated while free monoamines are low.	DAT imaging shows virtually absent striatal uptake, whereas MRI is normal or displays only subtle changes. Levodopa and standard dopaminergic therapy are largely ineffective in DAT deficiency, while VMAT2-related disease may respond better to dopamine agonists than to levodopa.
Mitochondrial disorders (e.g., pyruvate carboxylase, respiratory chain defects, early-onset WARS2)	Infantile hypokinetic–rigid syndrome with hypomimia and axial hypotonia within a multisystemic context, including lactic acidaemia, failure to thrive or organ involvement.	Elevated lactate in blood and/or CSF, respiratory chain defects in muscle biopsy, sometimes with low CSF HVA; additional metabolic markers depend on the specific mitochondrial defect.	MRI frequently demonstrates leukoencephalopathy or cortical atrophy with relative sparing of basal ganglia in some cases. Levodopa is usually of limited benefit, and management focuses on targeted metabolic therapies (cofactors, vitamins, energy support), with early levodopa responsiveness and subsequent decline described in some WARS2-related cases.

Furthermore, very early-onset mitochondrial diseases (e.g., *PC*, *DNM1L*, *FARS2*), may exhibit infantile parkinsonism features with elevated lactate (1, 5, 6). Finally, secondary causes such as severe hypoxic–ischemic injury and encephalitis also need to be considered.

Treatment in this age group depends on the underlying etiology. AR GTPCH1, PTPS, SR, and TH deficiencies, and some DHPR defects show an excellent and sustained response to levodopa (with or without 5-HTP, BH4, and Monoamine Oxidase B (MAO-B) and/or Catechol-O-Methyltransferase (COMT) inhibitors). Early initiation of treatment may result in meaningful neurodevelopmental improvement (1, 4–6).

In AADC deficiency, only a minority of patients exhibit significant improvement with dopaminergic agonists, and gene therapy is available in selected cases (1, 4, 5). In DAT defects and *SLC18A2-*related disorders, response to levodopa or dopaminergic agonists is poor or absent (1, 4, 6). In mitochondrial diseases (*PC*, *DNM1L*, *FARS2*), L-dopa therapy shows limited benefit and treatment should be directed at the specific metabolic defect (1, 5, 6).

### 5.2 Childhood: 3–12 years

In childhood, the most common presentation of PP is a complex parkinsonian syndrome associated with additional movement disorders, including dystonia, chorea, and myoclonus. Concomitant epilepsy, ataxia, cognitive impairment or behavioral abnormalities, as well as signs of systemic involvement, are also frequently observed (1, 4, 6). Prevalent etiologies include neurotransmitter disorders—such as autosomal dominant *GCH1* (Segawa disease), *TH*, *SR*, selected *PTPS* deficiencies, and dopamine transporter (DAT) defects (1, 4–6, 26), —as well as NBIA disorders, including PKAN (*PANK2*), PLAN (*PLA2G6*), MPAN (*C19orf12*), and Kufor–Rakeb syndrome (*ATP13A2*) (2–4, 10). Additional causes include disorders of manganese metabolism (*SLC30A10*, *SLC39A14*), lysosomal storage disorders and other inborn errors of metabolism (such as *CLN2/3/6*, GM1 gangliosidosis type 3, and Niemann–Pick disease type C), selected mitochondrial disorders (e.g., *POLG*), and monogenic forms of juvenile parkinsonism (*ATP1A3* [RDP], *PRKRA*, *FBXO7*, *DNAJC6*, *SYNJ1*, *PINK1*, *PARK7*, *SPG11/15*, *SCA2/3*, *TAF1* [DYT3], *VPS13C*, *PTPA*, among others). Additional metabolic and neurodegenerative conditions (including mitochondrial diseases and leukodystrophies), as well as acquired causes such as hypoxic injury, infections, and exposure to dopamine-blocking drugs, should also be considered (1, 3–6, 27) (see Table 4).

**Table 4:** Childhood parkinsonism (3–12 years): clinical features and diagnostic workup (4, 5, 25, 26).

Etiologic group/examples	Key clinical features	Core biomarkers	Typical imaging/response to treatment
Dopamine synthesis defects with childhood onset (dominant GCH1/Segawa, TH type A, SR deficiency)	Childhood-onset lower-limb dystonia with equinus gait and marked diurnal fluctuation, sometimes evolving to parkinsonism; milder hypokinetic–rigid presentations with gait disturbance or coarse tremor, oculogyric crises, developmental delay and occasional ataxia.	CSF HVA and 5-HIAA ranging from slightly reduced to clearly low, with normal or mildly altered pterins in dominant GCH1 and TH type A; SR deficiency shows low HVA/5-HIAA with elevated CSF sepiapterin and biopterins.	Brain MRI is usually normal or shows only mild atrophy; very low doses of levodopa often induce a striking, sustained response in Segawa syndrome, while TH type A and SR deficiency also respond well to levodopa (frequently combined with BH4 and MAO-B inhibitors), at the expense of possible dyskinesias in some patients.
Neurodegeneration with brain iron accumulation (PANK2/PKAN, PLA2G6-related disorders, C19orf12/MPAN, WDR45/BPAN)	Early or mid-childhood onset of severe dystonia with dysarthria and pyramidal signs, or psychomotor regression, ataxia and neuropathy; many patients later develop juvenile dystonia–parkinsonism with cognitive decline or epilepsy, and deterioration is typically progressive.	No single specific blood biomarker is consistently available; serum ferritin is often normal, and only non-specific markers (such as lactate or axonal injury markers) may be abnormal in some PLA2G6-related cases.	MRI classically shows the “eye-of-the-tiger” sign in the globus pallidus in PKAN, cerebellar atrophy with pallidal iron in PLA2G6-related disease, T2 hypointensity of globus pallidus greater than substantia nigra with mild cerebral or cerebellar atrophy in MPAN, and a T1 hyperintense halo in substantia nigra with T2 hypointensity in globus pallidus/substantia nigra in BPAN; levodopa response is absent or modest and usually transient, so management is mainly symptomatic, including baclofen, botulinum toxin, tetrabenazine and deep brain stimulation in selected cases.
Juvenile complex dystonia–parkinsonism due to ATP13A2 and related genes	Early adolescence or late childhood onset of dystonia–parkinsonism with pyramidal signs and, in ATP13A2, supranuclear ophthalmoplegia; a broader spectrum of juvenile dystonia–parkinsonism with cognitive decline and sometimes ophthalmoplegia is seen across ATP13A2, PLA2G6 and FBXO7.	No reliable peripheral biomarker; diagnosis relies on genetic testing, sometimes supported by non-specific mitochondrial or axonal markers.	MRI may show cortical, cerebellar and brainstem atrophy with T2 hypointensity of basal ganglia in ATP13A2 and additional iron-accumulation signs in PLA2G6. Levodopa often produces an initial clinical benefit but motor complications and progressive disability typically emerge early, making long-term management largely symptomatic.
Lysosomal and storage disorders presenting with juvenile parkinsonism (NPC1/2, GLB1-related GM1 type 3, neuronal ceroid lipofuscinoses CLN2/3/6)	Combination of ataxia, dystonia-parkinsonism, vertical supranuclear gaze palsy and organomegaly in Niemann–Pick type C; in GM1 type 3, dystonia-parkinsonism with dysarthria, short stature and skeletal dysplasia; in juvenile neuronal ceroid lipofuscinoses, epilepsy, regression and ataxia with gait freezing or parkinsonism in later stages.	Disease-specific biomarkers include positive filipin staining and elevated plasma oxysterols in NPC; reduced leukocyte β-galactosidase activity in GM1; and characteristic lysosomal enzyme deficiencies or pathogenic CLN variants in neuronal ceroid lipofuscinosis.	MRI typically demonstrates cerebellar with or without supratentorial atrophy in NPC, bilateral T2 hyperintensity of the putamen in GM1, and combined cerebral and cerebellar atrophy in CLN disorders; miglustat is available as a specific therapy for NPC, enzyme replacement is approved for CLN2 in some countries, and levodopa or dopaminergic agonists may provide only limited or gait-focused benefit.
Mitochondrial and respiratory chain disorders (e.g., POLG, mitochondrial RC defects)	Childhood-onset parkinsonism-plus with ataxia, peripheral neuropathy and ophthalmoplegia, often accompanied by seizures or other multisystem features, typically appearing in mid- to late childhood.	Elevated lactate in blood and/or CSF, respiratory chain defects in muscle biopsy, sometimes with low CSF HVA; additional metabolic markers depend on the specific mitochondrial defect.	MRI frequently shows cerebral or cerebellar atrophy and lesions in the basal ganglia or cerebellum, while dopaminergic SPECT or PET reveals reduced presynaptic uptake; levodopa and dopaminergic agonists can be transiently helpful but often precipitate dyskinesias, and treatment is predominantly supportive and focused on mitochondrial care.

Treatment in this age group is heterogeneous. Dominant GCH1 (Segawa disease) and other BH4/TH/SR deficiencies typically show an excellent and sustained response to levodopa (5, 6). In NBIA disorders, response to levodopa is variable and usually partial, so symptomatic treatment with tetrabenazine, baclofen, botulinum toxin and, in selected cases such as PKAN, deep brain stimulation (DBS) is often required (1, 4, 6). In Hypermanganesemia with dystonia, types 1 and 2, levodopa is generally ineffective and chelation therapy with disodium calcium edetate (Na₂CaEDTA) is the mainstay of treatment (1, 6, 20). In lysosomal diseases and other inborn errors of metabolism (IEMs), levodopa benefits are usually limited, and therapy should be focused on underlying etiologies. In juvenile monogenic parkinsonism, *PINK1*, *DJ-1* and *PRKN* mutations are associated with an excellent response to L-dopa but also with a high rate of early fluctuations and dyskinesias (3, 4, 6). In ATP1A3-related disease, the response to L-dopa is poor (1, 3, 6), and in *PRKRA*, *FBXO7*, *DNAJC6*, *SYNJ1*, *SPG11* and *SPG15*, L-dopa may improve bradykinesia but rarely normalizes motor function and is often associated with early complications (1, 3, 4, 6).

### 5.3 Adolescence: 13–21 years

The syndrome often presents as “typical” juvenile parkinsonism in adolescence. Bradykinesia, rigidity and resting tremor are predominant, and foot dystonia is also common. L-dopa treatment frequently leads to fluctuations and dyskinesias (3, 4, 6). In more complex presentations, additional signs such as pyramidal features, ataxia, neuropathy, cognitive decline, epilepsy or psychiatric manifestations may be present (1, 3, 4, 6). Etiologies are predominantly genetic and include autosomal recessive, autosomal dominant and X-linked conditions (see Table 5).

**Table 5:** Adolescence parkinsonism (13–21 years): clinical features and diagnostic workup (4, 5, 25, 26).

Etiologic group/examples	Key clinical features	Core biomarkers	Typical imaging/response to treatment
Monogenic juvenile parkinsonism with predominant nigrostriatal degeneration (PRKN, PINK1, PARK7/DJ-1)	Classic juvenile parkinsonism with bradykinesia, rigidity and foot dystonia, usually with a slowly progressive course; psychiatric or pyramidal signs may accompany PINK1 or DJ-1 variants.	No specific peripheral biomarker; diagnosis relies on genetic testing.	Brain MRI is normal or shows only mild atrophy, whereas dopaminergic SPECT or PET reveals a clear presynaptic deficit; levodopa produces an excellent and sustained response but is frequently complicated by early motor fluctuations and dyskinesias, for which dopamine agonists, MAO-B inhibitors, amantadine and, in selected cases, deep brain stimulation are useful.
Complex dystonia–parkinsonism with additional pyramidal/cognitive features (ATP13A2, PLA2G6, FBXO7)	Juvenile-onset dystonia–parkinsonism with pyramidal signs and cognitive decline, sometimes with supranuclear ophthalmoplegia or other oculomotor abnormalities.	No robust disease-specific peripheral biomarker; genetic confirmation is required, occasionally supported by non-specific metabolic or axonal injury markers.	MRI often shows cerebral and cerebellar atrophy, with imaging signs of neurodegeneration with brain iron accumulation in PLA2G6 and basal ganglia hypointensity in ATP13A2. Levodopa usually provides only a moderate initial benefit, followed by rapid progression and complications, so long-term management is predominantly symptomatic and may include deep brain stimulation.
Rapid-onset or stress-triggered dystonia–parkinsonism (ATP1A3/DYT12, PRKRA/DYT16)	Childhood or adolescent dystonia–parkinsonism of abrupt onset, often precipitated by fever or exertion in ATP1A3, or rapidly developing juvenile dystonia–parkinsonism in PRKRA; axial parkinsonism and pyramidal signs are common.	No specific biochemical signature; diagnosis is based on genetic testing.	MRI is normal or shows only non-specific changes. Levodopa may improve bradykinesia but usually has limited impact on dystonia, and supportive, multidisciplinary management is required.
Synaptic and dopamine-related parkinsonism genes (DNAJC6, SYNJ1, DNAJC12, RAB39B)	Juvenile parkinsonism frequently accompanied by dystonia, epileptic seizures or cognitive impairment, depending on the gene; RAB39B typically presents as X-linked intellectual disability with macrocephaly and Parkinson disease-like parkinsonism in adolescence or early adulthood.	DNAJC12-related disease may show low CSF HVA and other dopaminergic abnormalities, whereas DNAJC6, SYNJ1 and RAB39B are primarily defined by their genetic findings.	MRI can be normal or reveal cortical and/or basal ganglia atrophy; dopaminergic imaging (when performed) supports a presynaptic deficit. Levodopa generally elicits a good initial response, although the course is progressively degenerative.
Hereditary chorea-parkinsonism and spinocerebellar ataxias (HTT juvenile Huntington, ATXN2/ATXN3 SCA2/3)	Juvenile Huntington disease combines chorea with dystonia–parkinsonism and prominent cognitive and psychiatric decline, whereas SCA2/3 may manifest cerebellar ataxia with pyramidal signs and parkinsonism in a subset of patients.	Expanded CAG repeats in HTT or in ATXN2/ATXN3 confirm the diagnosis.	Imaging shows marked caudate–putamen atrophy in juvenile Huntington disease and cerebellar plus brainstem atrophy in SCA2/3. Levodopa can alleviate bradykinesia in some patients, but overall management is mainly symptomatic, including neuroleptics or VMAT2 inhibitors (e.g., tetrabenazine) for hyperkinetic movements.
Juvenile metabolic disorders with parkinsonism (Wilson disease/ATP7B, cerebrotendinous xanthomatosis/CYP27A1, homocystinuria, late-onset NPC, hypermanganesemia SLC30A10/SLC39A14, untreated PKU)	Parkinsonism emerges within a multisystemic context that may include hepatic disease, tendon xanthomas, thromboembolic events, neuropsychiatric manifestations or long-standing metabolic abnormalities.	Each disorder has a characteristic biochemical profile, such as abnormal copper and ceruloplasmin in Wilson disease, elevated cholestanol in CTX, increased plasma homocysteine in homocystinuria, raised plasma phenylalanine in PKU or high plasma manganese in hypermanganesemia, alongside disease-specific genetic testing.	MRI patterns range from basal ganglia lesions in Wilson disease to cerebellar and dentate changes in CTX or T1 hyperintensity of basal ganglia in manganese accumulation. Targeted aetiological therapies (copper chelators, chenodeoxycholic acid, vitamin supplementation, phenylalanine-restricted diet, manganese chelation) are central, while levodopa is usually of secondary or limited efficacy.

Autosomal recessive causes include *PRKN*, *PINK1*, *DJ-1*, *ATP13A2*, *PLA2G6*, *FBXO7*, *DNAJC6*, *SYNJ1*, *SPG11* and *ZFYVE26* (SPG15) (3, 4, 6). Autosomal dominant genes and repeat expansion disorders include mutations in *ATXN2* (SCA2), *ATXN3* (SCA3), *TMEM240* (SCA21), *MAPT*, *C9orf72, CSF1R* and HTT (Westphal variant Huntington’s Disease). On the other hand, X-linked causes include mutations in *RAB39B*, *TAF1* (DYT3), *MECP2* (in males), *ATP6AP2*, *PGK1* and *BTK* (1, 3, 6).

In *PARK-parkin*, *PARK-PINK1*, and *PARK-DJ-1*, patients typically show an initially good response to levodopa; however, a very high incidence of early motor fluctuations and levodopa-induced dyskinesias is observed. This often necessitates the early use of dopamine agonists, amantadine, MAO-B inhibitors, and, in selected cases, deep brain stimulation (DBS). In *ATP13A2*-, *PLA2G6*-, and *FBXO7*-associated disorders, an initial response to levodopa may also be observed, although management must additionally address cognitive impairment, depression, and other associated symptoms, with DBS considered in selected patients (3, 4, 6). Disorders associated with *HTT*, *ATXN2*, *ATXN3*, *RAB39B*, *NOTCH2NLC* (neuronal intranuclear inclusion disease), *SPG11*, and *ZFYVE26* mutations typically represent broader neurodegenerative syndromes in which levodopa has limited efficacy. In juvenile-onset inborn errors of metabolism—such as Wilson disease, cerebrotendinous xanthomatosis (CTX), homocystinuria, late-onset Niemann–Pick disease type C, hypermanganesemia, and untreated phenylketonuria—levodopa is usually of limited benefit, and treatment should be directed toward the underlying disorder (1, 5, 6). In Wilson’s disease, the cornerstone of management is decoppering therapy, using chelating agents such as trientine and D-penicillamine, as well as zinc salts, with the aim of reversing symptoms in affected individuals or preventing disease onset in presymptomatic patients (3).

## 6 Diagnostic workup

### 6.1 Clinical assessment

The Infantile Parkinsonism-Dystonia Rating Scale (IPDRS) has recently been developed. It consists of 28 items grouped into three subscales: (I) non-motor symptoms, (II) motor symptoms and (III) dyskinesia. This scale was preliminarily validated by international experts in pediatric movement disorders and may be useful for assessing parkinsonism in children in clinical practice (28).

### 6.2 Laboratory

Across all age groups, work-up should begin with a broad basic laboratory profile aimed at identifying treatable and secondary causes and guiding subsequent genetic testing. This includes complete blood count, ESR/CRP, electrolytes, renal and liver function tests, blood glucose, thyroid function, vitamin B12 and folate, lactate, ammonia, homocysteine and manganese. Copper studies (ceruloplasmin, serum copper and 24-h urinary copper), lipid profile and bile acids, as well as toxicology screening for metals and antidopaminergic drugs when indicated by the clinical context, are also recommended (6, 29–31).

Metabolic and CSF studies are fundamental in infants and young children, since several etiologies are treatable (32–34). CSF and urine biomarkers, including CSF monoamines (HVA, 5-HIAA), pterins, 3-OMD, neopterin and biopterin, as well as glycine, lactic acid and amino acids, are key for diagnosing defects in dopamine and other monoamine synthesis or recycling (TH, GCH1, SPR, AADC, DHPR, etc.) (33–35). These investigations are not only diagnostic but also allow differentiation between parkinsonism-dystonia due to monoamine synthesis defects (characteristic HVA/5-HIAA and pterin patterns) and dopamine transporter deficiency, in which an elevated homovanillic acid to 5-hydroxyindoleacetic acid ratio is observed (8, 25).

Additional tests include urinary organic acids, acylcarnitines and plasma and urinary amino acids, along with targeted studies for specific conditions such as lysosomal disorders.

### 6.3 Neuroimaging

Beyond excluding structural lesions, neuroimaging can provide important clues to specific etiologies (6, 29, 31, 33, 34, 36). Brain MRI with T1, T2, FLAIR, diffusion and, in particular, susceptibility-weighted sequences (SWI/T2*) is very useful in NBIA (iron deposition and the “eye-of-the-tiger” sign), Wilson disease (basal ganglia and brainstem changes), leukodystrophies and other neurodegenerative or metabolic conditions.

Functional neuroimaging (DaTSCAN or dopaminergic PET) is not used routinely, although it can provide highly valuable information in selected cases (23, 25, 31, 36). Dopaminergic functional imaging can aid in distinguishing disorders with preserved nigrostriatal function from those characterized by nigrostriatal denervation (6).

### 6.4 Genetic studies

Genetic analysis is currently performed as a definitive test to confirm the diagnosis and identify the causative gene. However, a thorough clinical, laboratory and imaging evaluation aiming at excluding acquired causes is crucial before genetic testing. This assessment should guide the selection of the most appropriate genetic strategy, in order to avoid unnecessary tests and improve results interpretation. The selection of genetic tests depends of the age at onset, biomarker results indicating a specific etiologic group (monoamine disorders, NBIA, IEM, WD, etc.) and the complexity of the phenotype. Targeted gene panels are usually chosen according to the clinical, laboratory and neuroimaging phenotype, while exome or genome sequencing is reserved for patients with negative panel testing or very complex phenotypes (4, 6, 31, 37–39).

## 7 Discussion

This narrative review highlights PP as a rare, clinically heterogeneous, and predominantly genetic hypokinetic–rigid syndrome that differs fundamentally from adult Parkinson’s disease in its pathophysiology, phenotype, and clinical trajectory. In contrast to adult PD, the phenotype in pediatric patients is frequently incomplete and clinically complex, and its expression may evolve over time in relation to neurodevelopment. Phenotypical spectrum ranging from parkinsonism–dystonia and complex movement disorder phenotypes in infancy and early childhood to more “pure” monogenic parkinsonism in adolescence (1, 3, 4). We emphasize the broad etiological spectrum, including neurotransmitter disorders, inborn errors of metabolism, mitochondrial and lysosomal diseases, metal accumulation disorders, monogenic juvenile parkinsonism genes and acquired conditions. Importantly, several of these conditions are potentially treatable, underscoring the critical role of early recognition and structured diagnostic evaluation. The integration of clinical features, neuroimaging, biochemical markers, and early genetic testing is central to optimizing diagnostic yield and patient outcomes.

The main strength of this review lies in the comprehensive integration of clinical, pathophysiological, and etiological aspects of pediatric parkinsonism across the full pediatric age spectrum. By adopting an age-stratified and mechanism-based approach, it provides a clinically oriented framework that facilitates differential diagnosis and highlights potentially treatable conditions. In addition, the discussion of diagnostic tools—including the Infantile Parkinsonism–Dystonia Rating Scale (IPDRS) and the role of advanced neuroimaging, biochemical, and genetic testing—enhances the practical relevance of the manuscript for clinicians involved in pediatric movement disorders.

Also, this work has limitations inherent to its narrative design. The literature search was not conducted as a formal systematic review, and therefore some relevant studies may not have been captured and selection bias cannot be excluded. The low epidemiological prevalence and the heterogeneity of PP limit the availability of large, controlled studies, and much of the existing evidence is gathered from case series and observational reports. Furthermore, genotype–phenotype correlations and long-term outcome data remain incomplete for many etiologies, which constrains definitive conclusions regarding prognosis and optimal management strategies.

Important knowledge gaps remain in pediatric parkinsonism, particularly regarding its true epidemiological burden, natural history, genotype–phenotype correlations, and long-term treatment outcomes across different etiologies. Standardized diagnostic criteria and validated pediatric-specific clinical rating scales are still limited, which complicates both early diagnosis and longitudinal follow-up. In addition, reliable biomarkers for disease progression and treatment response are lacking, and evidence supporting disease-modifying therapies remains scarce for most conditions. Addressing these gaps will be essential to improve diagnostic accuracy, prognostic stratification, and personalized therapeutic approaches.

Future research should focus on the establishment of multicenter, longitudinal cohorts to better define the natural history and treatment response of pediatric parkinsonism across different etiologies. Epidemiological studies addressing the prevalence and incidence of pediatric parkinsonism are also needed. Advances in genomic technologies, multi-omics approaches, and biomarker discovery are expected to improve diagnostic accuracy and enable earlier identification of treatable disorders. The development and validation of standardized clinical scales and diagnostic algorithms tailored to different developmental stages will be essential. Finally, expanding access to disease-modifying therapies—including targeted metabolic treatments, gene therapy, and neuromodulation—represents a key priority to improve long-term outcomes in this vulnerable population.

## 8 Conclusion

PP is a rare hypokinetic–rigid syndrome that is clinically heterogeneous and predominantly genetic in origin, and it differs substantially from adult Parkinson’s disease with respect to pathophysiological mechanisms, clinical phenotype, and disease course. Clinical manifestations are strongly influenced by neurodevelopmental stage, with parkinsonism–dystonia phenotypes predominating in infancy, complex movement disorder presentations during childhood, and monogenic “pure” parkinsonism emerging more often in adolescence.

Given the broad etiological spectrum and the existence of potentially treatable causes, a structured diagnostic approach integrating age at onset, clinical features, laboratory studies, and neuroimaging—together with early implementation of comprehensive metabolic and genetic testing—is essential to optimize diagnosis and prognosis. The use of disease-specific assessment tools, such as the Infantile Parkinsonism–Dystonia Rating Scale, and practical clinical algorithms may further support standardized evaluation, monitoring of disease progression, and informed therapeutic decision-making, including rational use of levodopa, targeted metabolic therapies, and neuromodulation in selected cases.
